# Surgery

**Published:** 1911-03

**Authors:** Robert G. Poole Lansdown


### SURGERY.

**Pneumococcal arthritis.**—In a valuable paper Campbell Howard [1] deals fully with this affection, and gives a résumé of the literature of the subject, to which I am indebted for most of my references.

The presence of a diplococcus in joint effusions occurring as a sequel of croupous pneumonia was first described by Foa and Bordoni-Uffreduzzi [2] in 1888, while the first fully reported case was published by Weichselbaum [3] later in the same year, in a paper entitled, "On unusual localisation of the Pneumonic Virus."

Before this date French observers had pointed out that croupous pneumonia was complicated by inflammation of joints.

An excellent paper published in 1901, entitled "Pneumococcal Arthritis," by Cave, [4] of Bath, appears to be the first contribution of any importance published on the subject in this country. Pneumococcal arthritis is undoubtedly a rare condition, but with the increase of specialism and the multiplication of bacteriological laboratories taking place at the present day, the number of cases reported is increasing year by year.

In 1899 Leroux [5] collected six instances from 4,256 cases of pneumonia. Cave, before his paper was published in 1901, could only find thirty-one cases, whereas Herzog, [6] up to 1906,

[1] Howard, *Johns Hopkins Hosp. Rep.*, 1910, xv. 229.

[2] Foa and Bordoni-Uffreduzzi, *Ztschr. f. Hyg.*, 1888, iv. 67.

[3] Weichselbaum, *Wien. med. Wchnschr.*, 1888, xxxviii. 1177, 1209.

[4] Cave, *Lancet*, 1901, i. 82.

[5] Leroux, *Thèse de Paris*, 1900, 137.

[6] Herzog, *Jahrb. f. Kinderh.*, 1906, lxiii. 446.

had collected ninety-one, to which collection Howard[1] adds three other cases, reported in 1906 by Secretan and Wrangham,[2] Raw,[3] and Middleton.[4] As far as our present knowledge goes, pneumococcal arthritis undoubtedly affects males much more commonly than females—in 1901, at the time of Cave's paper, in the proportion of twenty-five to three, while Herrick,[5] in 1902, records forty males to seven females.

When arthritis becomes purulent in children up to six months, it is stated by Dudgeon and Branson[6] to be almost invariably due to the diplococcus of pneumonia, and Herzog mentions the frequency of pneumococcal arthritis in infancy.

Howard says that "we can no longer consider pneumococcic arthritis more common in adult life than in infancy, though we are not yet warranted in assuming the contrary."

The last-named writer classifies pneumococcal arthritis under three headings :—

I. *Meta-pneumonic* cases.—Those in which the joint affection occurs as a complication or sequel of acute lobar or lobular pneumonia.

II. *Præ-pneumonic* cases.—Those in which the joint affection precedes the pneumonia.

III. *Non-pneumonic* cases.—Those in which the joint affection occurs without any associated pneumonic inflammation of the lung tissue.

Class I is by far the commonest. Cave's figures work out at 90.3 per cent., Pfisterer's[7] 86.6 per cent., and Herzog's 78 per cent.

Class II is questioned by Howard, though Herzog collected four cases from the literature and added one of his own, making the percentage 5.5.

In Class III Herzog's figures give 16.4 per cent.

Howard shows that Classes II and III are more common relatively and absolutely in infancy and childhood than in adult life, during which period they occur but rarely.

In the meta-pneumonic cases the pneumococcus gains entry to the system undoubtedly through the respiratory tract. In cases occurring in children belonging to the præ-pneumonic and non-pneumonic groups, Herzog believes that the pneumococcus enters most commonly by a diseased middle ear, and the evidence he quotes in favour of his contention is very impressive.

In the majority of the meta-pneumonic cases the organism undoubtedly reaches the affected joint by the blood stream.

[1] Howard, *Johns Hopkins Hosp. Rep.*, 1910, xv. 229.
[2] Secretan and Wrangham, *Brit. M. J.*, 1906, i. 915.
[3] Stanley Raw, *Ibid.*, 1906, i. 1400. [4] Middleton, *Ibid.*, 1906, ii. 197.
[5] Herrick, *Am. J. M. Sc.*, 1902, cxxiv. 12.
[6] Dudgeon, *Lancet*, 1903, ii. 266. [7] Pfisterer, *Jahrb. f. Kinderh.*, 1902, lv.

Pfisterer mentions seven cases of unilateral pneumonia, in all of which the shoulder-joint and in three the sterno-clavicular joint were affected on the same side as the pneumonia, in support of the suggestion that in some cases the lymphatic system is responsible for the conveyance of the pneumococcus from the primary focus. Pneumococcal arthritis much more commonly affects one joint only than two or more joints. The larger joints seem to be more commonly affected than smaller ones. This one would naturally expect to be the case, as the larger the joint the greater its risk of traumatism, a rule too which holds good in other infections of joints superimposed upon previous traumatism.

The joints which have been most commonly reported as being affected are the shoulder and the knee. Herzog records finding four elbows and two ankles, and Herrick eight sterno-clavicular joints. Fells[1] reports at some length a case involving the left hip in a lad aged fifteen, which was operated upon by the writer of this article.

It is a well established fact that traumatism or previous disease of the joint play an important part as predisposing factors in pneumococcal arthritis. The writings of Leroux, Cave, Cole,[2] Pfisterer and Herrick show that in at least 31.41 per cent. of cases collected by them there was a note of either traumatism or disease of the joint preceding the onset of the pneumococcal affection. In Fell's case, in which the hip was affected, there was a definite statement that that particular joint had been sprained five days before the onset of pneumonia. In another case, operated upon by the writer and referred to later, there was definite evidence of the left shoulder being strained while the patient was being moved in bed during the course of the pneumonia.

The fact that trauma plays so important a part in predisposing to joint affections in pneumonia probably accounts for the much greater frequency of incidence in the male sex, men in the pursuit of their livelihood being much more exposed to injury than is the opposite sex. It may also account to some extent for the relative frequency of joint affections in early childhood. In this respect trauma plays the same prominent part in predisposing to the incidence of pneumococcal arthritis as it does in many other conditions in which the joints are liable to become subject to bacterial infection. Two common examples will suffice, gonococcal arthritis and tuberculous arthritis, in both of which affections it is the exception not to be able to obtain a definite history of strain or sprain or other previous injury to the diseased joint or joints.

[1] Fells, *Bristol M.-Chir. J.*, 1909, xxvii. 336.

[2] Cole, *Am. Med.*, 1902, iii. 905.

The nature of the fluid contained in the affected joints varies with the period of the disease at which the interior of the joint is inspected, from an excess of synovial fluid to pure pus. This is well borne out in the two cases with which the writer has been connected. In that reported by Fells, pain in the hip was complained of on the twenty-second day. When the joint was opened eleven days later the synovial membrane was much injected, and contained blood-stained synovial fluid, no fibrin and no pus. A culture made from this fluid at the time the joint was opened resulted in a pure growth of the diplococcus of pneumonia, an important point unintentionally omitted in the original report of the case. Five days later pus loaded with pneumococcus discharged from the wound; it ceased to discharge seven days later, and the wound healed. Twelve months later, when seen by the writer, the function of the joint was perfect.

The other case was a male, aged 51, who contracted right-sided pneumonia the last week of November, 1910. On December 13th pleurisy supervened at the right base, and on the same day he complained that his left shoulder was strained by his being pulled up in bed. Four days later a large pneumococcal empyema was opened on the right side. Pain was repeatedly complained of in the left shoulder, until on January 10th, 1911, it began to swell. On January 13th, thirty-one days after the first complaint of pain in the shoulder, an exploring needle, followed by a small incision under a local anæsthetic, voided several ounces of pure, odourless pus; a short drainage tube was inserted, relief from pain was immediate. The pus withdrawn gave a pure culture of the diplococcus pneumoniæ. Three weeks later the discharge had ceased and the wound was healed, but there was coarse friction felt on movement of the head of the humerus.

Again, in a case reported by Howard,[1] the patient complained on the twelfth day after onset of pneumonia of pain on movement or manipulation of the left ankle, and on inspection it was found to be red and swollen. On the twentieth day of the illness an exploring needle was inserted, and a syringeful of yellowish, purulent, blood-stained fluid was removed, which contained numerous pus cells and blood corpuscles, and which on culture gave a pure growth of diplococcus pneumoniæ. The joint was opened the same day, and the patient was discharged three and a half weeks later quite well, save for some swelling of the ankle.

Howard states that when recovery takes place ankylosis of the affected joint usually results. The prognosis in this respect is probably much influenced by the period at which, during the progress of the arthritis, the joint is opened.

[1] *Loc. cit.*

If the joint is opened and drained before the formation of pus, there seems to be less likelihood of subsequent ankylosis, and the shorter the length of time before suppuration ceases and the small incision has healed.

The joint should be opened with the most rigid aseptic precautions under a local anæsthetic, and a culture should always be made from the contained fluid. It is probably wise, if the patient's condition allows, to thoroughly wash out the joint cavity with sterilised normal saline at the body temperature, after inserting a small drainage tube. A bandage or strapping should be so arranged over the sterile dressing as to prevent the retention of secretion in the normal pouches of synovial lining; the limb should be so arranged as to immobilise the affected joint, and if that joint be the shoulder, in such manner as shall not impede the movements of the chest wall during respiration. It is possible that considerable assistance in resolution is derived from administering in suitable dosage vaccines manufactured from the cultures obtained from the joint cavity before removal of the drainage tube.

In the case related above the improvement noticed, not only in the shoulder-joint but also in the lung and empyema wound, after the administration of vaccines was marked.

In such joints as the elbow, knee and ankle much benefit might also be derived from the application of elastic bandages to produce hyperæmic congestion of the joint after the manner advised by Bier.

ROBERT G. POOLE LANSDOWN.

